# Supplementary material for: The geographical pension gap: Understanding the causes of inequality in China’s pension funds
Source: PLoS One. 2023 Jul 17;18(7):e0288754. doi: 10.1371/journal.pone.0288754 (PMC10351713; doi:10.1371/journal.pone.0288754)
Supplement: Attachment — Submitted filename: Response to Reviewer 2 Comments.docx [file pone.0288754.s001.docx]

**Response to Reviewer 2 Comments**

Dear reviewer,

We express our heartfelt gratitude to the reviewer for thoroughly examining our manuscript and offering constructive comments that guided our revision process. We have diligently incorporated your valuable suggestions into the manuscript, and we appreciate your time and effort in reviewing our work. Please find detailed responses to your comments and suggestions below. Once again, we extend our sincere thanks to the reviewer for their invaluable contribution.

（1）China has seen a sharp reduction of poverty, but also a substantial increase of inequality. As the result of more than two decades of rapid economic growth in China, millions have been lifted out of poverty, resulting in an impressive decline in the poverty . I will urge authors to write more about literature and focus on the gap.

Thank you for your suggestion. Following your advice, we have incorporated the following content: *"Since implementing economic reforms and opening up, China has experienced rapid economic development and substantially reduced absolute poverty. However, the issue of relative poverty remains severe, and the widening income gap poses a significant obstacle to achieving common prosperity (1). Establishing the old-age insurance system has been a crucial policy tool to narrow this income gap. Nevertheless, China faces the unique challenge of “getting old before getting rich” due to the relatively short period during which the old-age security system has been in place and the accelerated population aging, particularly among certain demographic groups (2).*

*Initially, China’s pension insurance system was designed to safeguard the well-being of older adults, prevent elderly poverty, and reduce income disparities among older individuals. Currently, the pension insurance system in China has achieved nearly universal coverage among the population, with an increasing participation rate. However, due to inadequate overall planning of the pension insurance fund at the national level, a significant disparity exists in the income and expenditure of pension funds across different provinces. As a result, some provinces face shortfalls in pension fund revenue, resulting in a decline in the growth rate of the fund balance. Consequently, this not only negatively impacts efforts to alleviate poverty among older adults but also exacerbates regional income disparities among this age group, contradicting the original intent of the pension insurance system (3). Furthermore, demographic changes are generating global concerns regarding the long-term financial sustainability of pension schemes (4, 5, 6), a matter of particular urgency for China."*

Furthermore, we have included a comprehensive literature review section, which provides detailed insights within the manuscript.

（2）Kindly elaborate more about research methodology .

Thank you for your suggestion. Following your advice, we have incorporated the following content: *“In measuring regional disparities, numerous calculation methods have been utilized in previous studies, including the coefficient of variation, Theil index, standard deviation, and Gini coefficient. However, when evaluating changes in the absolute gap between pension regions, comparing the absolute gaps at different time points can be misleading due to variations in the balance growth rate and the overall balance level. To address this issue, the relative gap is employed in this study, which eliminates the direct impact of the total size and enables a more meaningful assessment of changes in regional disparities over time. In addition, the growth rate of fund balances in each region is calculated to reflect these changes.*

*Furthermore, China has historically exhibited significant disparities in fund balances among its Eastern, Central, Western, and Northeastern regions, primarily due to their differing geographical locations. The advantage of utilizing the Theil index lies in its ability to measure and compute gaps between and within regions, making it a suitable choice for quantifying the discrepancies in fund balances.*

*Lastly, this study adopts the geographic detector method to identify multiple influencing factors contributing to regional fund balance gaps. Unlike conventional spatial regression analysis, the geographic detector method does not rely on linear assumptions or conditional restrictions. It offers an objective means of determining the degree of interpretation of independent variables on the dependent variable and provides the added advantage of detecting interactions between these factors.”*

（3）Discussion part is not satisfactory.

We appreciate your suggestion. Taking your advice into account, we have made adjustments to the theoretical and practical contributions discussed in the manuscript's Discussion section. These revisions have been thoroughly elaborated upon within the manuscript itself.
